# Cadmium Stress Response of ABC Transporters in *Ligusticum chuanxiong*: Genome-Wide Identification and Bioinformatic Characterization

**DOI:** 10.3390/genes16101235

**Published:** 2025-10-18

**Authors:** Yun Zhen, Xiang Chen, Ruoshi Li, Shunlu Chen, Can Wang, Chi Song, Guihua Jiang, Xianmei Yin

**Affiliations:** 1 School of Pharmacy, Chengdu University of Traditional Chinese Medicine, Chengdu 611137, China; 2 Institute of Herbgenomics, Chengdu University of Traditional Chinese Medicine, Chengdu 611137, Chinasongchi@cdutcm.edu.cn (C.S.); 3 Innovative Institute of Chinese Medicine and Pharmacy, Chengdu University of Traditional Chinese Medicine, Chengdu 611137, China

**Keywords:** *Ligusticum chuanxiong* Hort., cadmium stress, ATP-binding cassette transporter family, qRT-PCR

## Abstract

**Background**: *Ligusticum chuanxiong* Hort. is a well-known traditional Chinese medicinal herb whose clinical application and international trade had been constrained by cadmium (Cd) contamination. However, the molecular mechanisms underlying its response to cadmium stress remained poorly understood. The ATP-binding cassette (ABC) transporter family plays crucial roles in various plant processes, including growth and development, hormone transduction, and stress responses. This study aimed to analyze the ABC transporter genes in *L. chuanxiong* to better understand their roles during cadmium stress responses. **Methods**: Genome-wide identification of *ABC* genes in *L. chuanxiong* was performed, and transcriptome sequencing of rhizomes under cadmium stress was conducted. Differentially expressed *LcABC* genes were screened using bioinformatic analysis. **Results**: A total of 368 *LcABC* genes were identified. Transcriptome analysis revealed 37 upregulated *LcABC* genes, which were classified into six subfamilies. Cis-element analysis indicated that their promoters contain hormone-, growth-, and stress-responsive elements. Notably, *LcABCG8*, *LcABCG48*, and *LcABCG108* contain stress-responsive elements and show close evolutionary relationships with heavy metal-responsive genes such as *AtABCC1/2/3* and *AtABCG36/40*, suggesting that they could be key candidates. qRT-PCR validation of nine *LcABC* genes confirmed their differential sensitivity to cadmium stress. **Conclusions**: This study conducted a comprehensive identification of the ABC gene family in *L. chuanxiong.* By integrating transcriptomic data with systematic bioinformatic analyses, we identified several LcABC transporters that may play important roles in cadmium stress responses. The results provide insights into the molecular mechanisms of ABC transporters in cadmium stress responses in *L. chuanxiong* and offer strategies for reducing cadmium accumulation.

## 1. Introduction

The dried rhizome of *L. chuanxiong* is one of the most extensively utilized traditional Chinese medicines. It exhibits considerable pharmacological activities in the cardiovascular, cerebrovascular, nervous, respiratory, hepatic, and renal systems [1]. Furthermore, *L. chuanxiong* is employed as a natural additive in cosmetics and is used in sachets and fragrances due to its distinctive volatile aroma, highlighting its significant medicinal value and substantial commercial potential. However, pronounced cadmium contamination in *L. chuanxiong* had resulted in recurrent export rejections and destruction, emerging as a critical barrier to international trade and concurrently impacting its clinical safety and efficacy [2,3]. Studies have demonstrated that cadmium uptake inhibits growth, development, and photosynthesis in *L. chuanxiong* and disrupts the biosynthetic metabolism of bioactive constituents such as ferulic acid and ligustrazine [4]. Conventional research on cadmium mitigation has primarily focused on agronomic soil remediation, employing practices such as fertilizer application [5], microbial agent amendments [6], and alterations to traditional cultivation models [7] to reduce cadmium accumulation in the rhizomes. These soil-focused strategies only address the external factors and the studies did not investigate the most crucial determinant of hyperaccumulation: the intrinsic cadmium enrichment mechanism of *L. chuanxiong* itself [8]; thus, there is inadequate data to guide sustainable resolution. Consequently, elucidating the molecular mechanisms of cadmium enrichment in *L. chuanxiong* is essential for fundamentally addressing the contamination issues. ATP-binding cassette (ABC) transporters, which belong to a superfamily of membrane proteins, are ubiquitous in both prokaryotes and eukaryotes [9]. These transporters had been implicated in regulating diverse plant processes including cuticular lipid transport [10], hormone translocation [11], vacuolar sequestration of toxic compounds [12], seed development [13], and plant adaptation [14]. A genome-wide analysis of ABC transporters in *Prunus persica* had identified *PpABCC1* as a gene involved in anthocyanin accumulation [15]. AtABCD1 had been recognized as a key protein for benzoic acid synthesis participating in plant–pathogen interactions [16]. The *CaABCG41* gene in chickpea had been characterized as a drought-responsive gene [17], while ABCG subfamily members in soybean had shown enhanced activity under cadmium and mercury stress [18]. Research on lentil had confirmed that *LcABCI14* and *LcABCI17* genes exhibited elevated expression under aluminum stress [19]. Collectively, these findings demonstrate the indispensable roles of ABC transporters in plant growth, development, and environmental adaptation.

Nevertheless, comprehensive identification and characterization of *ABC* gene family members in medicinal plants had remained relatively limited. Previous studies by Zhang [14] and Xie [20] had proposed ABC transporters as crucial carriers involved in cadmium transport in *L. chuanxiong*; however, a systematic analysis of the *ABC* gene family specific to this species had been lacking. Therefore, this study had identified 368 *ABC* genes from the *L. chuanxiong* genome and had selected 37 high-priority candidate genes through integration with cadmium stress transcriptome data. These candidates had subsequently undergone systematic analyses of physicochemical properties, conserved motifs, cis-acting regulatory elements, and expression patterns, thereby providing a valuable reference for further elucidating the molecular mechanisms underlying the cadmium stress response in *L. chuanxiong*.

## 2. Materials and Methods

### 2.1. Plant Materials and Treatments

We collected *L. chuanxiong* seedlings from the Medicinal Plant Garden at Chengdu University of Traditional Chinese Medicine (30°41′ N, 104°48′ E).

Uniformly developed 6-week-old seedlings were selected and transplanted into soil treated with CdCl_2_·2.5H_2_O solution. In this study, concentration gradients of 0, 80, 160, and 320 mg/kg were established to profile transcriptional responses across a progression of cadmium stress levels. This concentration range falls within the cadmium stress levels established by Yang Anfu et al., which have been demonstrated to effectively induce cadmium stress and evaluate plant responses [21]. All treatment groups were cultivated outdoors under rain-sheltered conditions for two weeks, with three biological replicates maintained per concentration. Excessive cadmium accumulation can induce various physiological impairments in plants, including growth retardation, reduced chlorophyll content, leaf chlorosis, and decreased photosynthetic rates [22]. Based on the methodology of Wang et al. [23] and combined with the observed chlorosis in our experimental plants, the two-week stress period was selected as the sampling time point. Rhizome samples of *L. chuanxiong* were then collected, flash-frozen in liquid nitrogen, and stored at −80 °C.

### 2.2. Identification and Chromosomal Distribution of LcABC Gene Family Members

The *L. chuanxiong* genome and structural annotation data were downloaded from the Figshare database (https://figshare.com/projects/Ligusticum_chuanxiong_genome/169136, accessed on 16 November 2024) [24]. The pfam-scan.pl script had been employed to scan sequences against the Pfam-A database to identify proteins containing characteristic PF00005, PF00664, PF12698, PF12848, PF01061, PF19055, PF08370, and PF14510 domains. Concurrently, the protein sequences of AtABC transporters had been retrieved from TAIR website (https://www.arabidopsis.org/, accessed on 15 January 2025). Reciprocal BLASTP analysis (E-value < 1 × e^−5^) was performed between the ABC transporter sequences of *A. thaliana* and *L. chuanxiong* to identify preliminary candidate genes. The candidate sequences were further validated using NCBI Protein BLAST (https://www.ncbi.nlm.nih.gov/, accessed on 16 January 2025), and those lacking characteristic ABC domains were excluded based on analysis via the Conserved Domain Database (CDD) (https://www.ncbi.nlm.nih.gov/cdd/, accessed on 16 January 2025). Finally, based on the sequence alignment results and phylogenetic analysis, the identified *ABC* gene family members in *L. chuanxiong* were classified into subfamilies and renamed accordingly. Chromosomal locations of these genes had been mapped and visualized using the GTF/GFF gene localization plugin in TBtools V2.056 software [25].

### 2.3. Analysis of Gene Duplication and Homology in the LcABC Gene Family

The genome and annotation files of both haplotypes of *L. chuanxiong* had been submitted to TBtools V2.056 [25] for collinearity analysis. The genomic chromosome sequences and structural annotation information for *Arabidopsis thaliana* (Taxonomy ID: 3702) and *Daucus carota subsp. sativus* (Taxonomy ID: 79200) had been downloaded from the Ensembl Plants Database (https://plants.ensembl.org/index.html, accessed on 20 January 2025). MCScanX analysis in TBtools V2.056 [17] had been employed to analyze gene duplication events among *ABC* gene family members in *L. chuanxiong*, *A. thaliana*, and *D. carota*. Collinear blocks with scores > 500.0 and collinear gene pairs with E-values < 1 × e^−50^ had been selected for subsequent schematic diagram construction.

### 2.4. RNA Isolation and Sequencing

Rhizomes of *L*. *chuanxiong* subjected to cadmium stress treatments at concentrations of 0, 80, 160, and 320 mg/kg had been collected, with three biological replicates established for each concentration level. Subsequent sequencing services had been entrusted to Shanghai Personal Biotechnology Co., Ltd. (Shanghai, China). The experimental procedure commenced with total RNA extraction from the tissues using TRIzol^®^ reagent (Invitrogen, Carlsbad, CA, USA), with subsequent digestion using DNase I (Takara Bio Inc., Shiga, Japan) to remove genomic DNA. RNA concentration and purity had been assessed using Thermo Scientific NanoDrop 2000 (Thermo Scientific, Waltham, MA, USA), followed by integrity assessment and quantification using an Agilent 2100 Bioanalyzer and the RNA 6000 Nano Kit 5067-1511 (Agilent Technologies Inc., Carlsbad, CA, USA). Total RNA samples meeting the quantity threshold of ≥1 μg had been selected for library preparation using the NEBNext Ultra II RNA Library Prep Kit for Illumina (New England Biolabs Inc., Ipswich, MA, USA). Poly(A)-tailed mRNA molecules had been enriched through oligo(dT) magnetic bead selection and subsequently fragmented via divalent cation-mediated disruption. First-strand cDNA synthesis had been performed employing fragmented mRNA as template with random oligonucleotide primers. Following double-stranded cDNA synthesis and purification, end repair procedures had been conducted, featuring 3′-end adenylation and sequencing adapter ligation. cDNA fragments ranging from 400 to 500 bp had been size-selected using AMPure XP beads, PCR-amplified, and repurified with AMPure XP beads to generate final sequencing libraries. Library quality assessment had been performed using the Agilent 2100 Bioanalyzer with the High Sensitivity DNA Kit 5067-4626 (Agilent Technologies Inc., Carlsbad, CA, USA). Total library concentration quantification had been carried out through Pico Green (Quantifluor-ST fluorometer, Promega, Madison, WI, USA, E6090; Quant-iT PicoGreen dsDNA Assay Kit, Invitrogen, Carlsbad, CA, USA, P7589), while effective library concentration had been determined via qPCR quantification (StepOnePlus Real-Time PCR System, Thermo Scientific, Waltham, MA, USA). Multiplexed DNA libraries had been normalized, pooled in equal volumes. After gradual dilution and quantification, sequencing had been performed on an Illumina platform in PE150 mode. The raw paired-end reads had been trimmed and quality-controlled with FastP (v0.22.0, default parameters). Using the *L. chuanxiong* reference genome and structural annotations downloaded from the publicly accessible Figshare repository (https://figshare.com/projects/Ligusticum_chuanxiong_genome/169136, accessed on 16 November 2024), we employed HISAT2 (v2.1.0) for index construction and alignment of paired-end clean reads.

### 2.5. Screening of Upregulated ABC Genes Under Cadmium Stress

To identify significantly up-regulated *ABC* gene family members under cadmium stress, this study had utilized gene expression data quantified in FPKM from both blank control groups without cadmium treatment and samples subjected to varying concentrations of cadmium stress. A threshold of log2 Fold Change > 0.5 and a statistical significance level of *p*-value < 0.05 had been established as the criteria. Differential expression analysis had been performed using the limma package [26] in R (V5801.9.0.0). Significantly up-regulated *LcABC* genes under cadmium stress had been subsequently screened as core targets for prioritized investigation.

### 2.6. Phylogenetic Analysis

Multiple sequence alignment had been performed using the MAFFT online server [27] (https://mafft.cbrc.jp/alignment/server/, accessed on 28 March 2025) for protein sequences of the up-regulated LcABC transporters along with ABC family members from *D. carota* and *A. thaliana*. The alignment files were uploaded to the ATGC PhyML 3.0 tool [28] (http://www.atgc-montpellier.fr/phyml/, accessed on 30 March 2025) where the SMS model selection method had been employed to automatically determine the optimal substitution model for phylogenetic tree construction. Following initial phylogenetic reconstruction, the exported tree files had been graphically refined and annotated using the ITOL online platform [29] (itol.embl.de/, accessed on 1 April 2025).

### 2.7. Physicochemical Characterization and Structural Prediction

Physicochemical property analysis of the up-regulated differentially expressed LcABC family members had been performed using the ExPASy online tool [30] (https://www.expasy.org/, accessed on 2 April 2025). Subcellular localization prediction had subsequently been conducted using the WoLF PSORT website [31] (https://wolfpsort.hgc.jp, accessed on 2 April 2025). Additionally, transmembrane helix prediction had been carried out through the DeepTMHMM-1.0 server [32] (https://services.healthtech.dtu.dk/services/DeepTMHMM-1.0, accessed on 2 April 2025) to characterize their transmembrane structural properties.

### 2.8. Motif, Domain, and Gene Structure Analysis

To gain deeper insights into the gene structures of the up-regulated differentially expressed LcABC transporters, conserved motifs within their protein sequences had been predicted using the MEME suite (https://doi.org/10.1093/nar/gkv416, accessed on 4 April 2025) with the target number of motifs set to 15. Subsequently, conserved domain analysis had been performed online using Batch CD-Search tool (https://www.ncbi.nlm.nih.gov/cdd/, accessed on 4 April 2025). Integrated with the genomic structural annotation information of *L. chuanxiong*, visualization and graphical refinement had been conducted employing the TBtools-Gene Structure View functionality [25].

### 2.9. cis-Regulatory Element Prediction

The promoter sequences (2000 bp upstream) of the up-regulated differentially expressed *LcABC* genes were extracted, and the results of the analyses were collated and simplified by predicting cis-acting elements of the target sequences via the online tool PlantCare (https://bioinformatics.psb.ugent.be/webtools/plantcare/html/, accessed on 8 April 2025). The processed data had finally been visualized using the TBtools V2.056 [25].

### 2.10. qRT-PCR Analysis

Total RNA was extracted from cadmium-stressed and control *L. chuanxiong* rhizomes and reverse transcribed into cDNA (R411-01, R323-01; Vazyme, Nanjing, China). Primers for qRT-PCR reaction were designed using NCBI Primer-BLAST (https://www.ncbi.nlm.nih.gov/tools/primer-blast/, accessed on 12 April 2025) and synthesized by Sangon Biotech (Shanghai, China) (Supplementary Table S1). The qRT-PCR reaction was assembled using a commercial kit (Q711-02, Vazyme, Nanjing, China), and quantitative real-time PCR was performed on a JLM-QX series real-time PCR system. Each sample was run in three technical and biological replicates. The Ct values were automatically determined by the JLM qPCR AnalyzerSoft with the threshold set uniformly within the exponential amplification phase for all reactions. The relative gene expression was calculated using the 2^−ΔΔCt^ method with *GAPDH* as the internal reference gene. Data statistics and visualization were conducted using GraphPad Prism (version 9.5).

## 3. Results

### 3.1. Identification and Chromosomal Distribution of LcABC Genes

**Figure 1:**
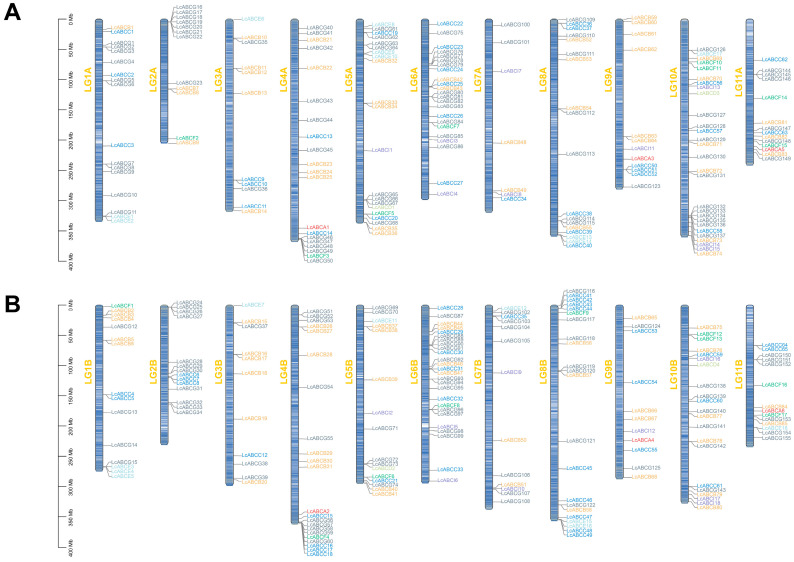
Chromosomal localization of *LcABC* genes. (**A**) Localization on Haplotype A chromosomes. (**B**) Localization on Haplotype B chromosomes. Each chromosome name is indicated on the left side of the respective blue bar, with gene names presented on the right in different color coded by the eight subfamilies. Scale marks indicate chromosome length (Mb).

A comprehensive analysis of the *L. chuanxiong* genome identified 368 *LcABC* genes, which were phylogenetically classified into eight subfamilies (ABCA-ABCG, ABCI) with widely varying sizes, ranging from 4 members (ABCD) to 155 (ABCG). Chromosomal localization of *LcABC* genes was visualized (Figure 1) based on genomic and annotation data. The 368 *LcABC* genes showed uneven distribution across the 22 chromosomes of two haplotypes. Both haplotypes exhibited clustered arrangements, suggesting tandem duplication events within the *LcABC* gene family.

### 3.2. Gene Duplication and Homology Analysis

**Figure 2:**
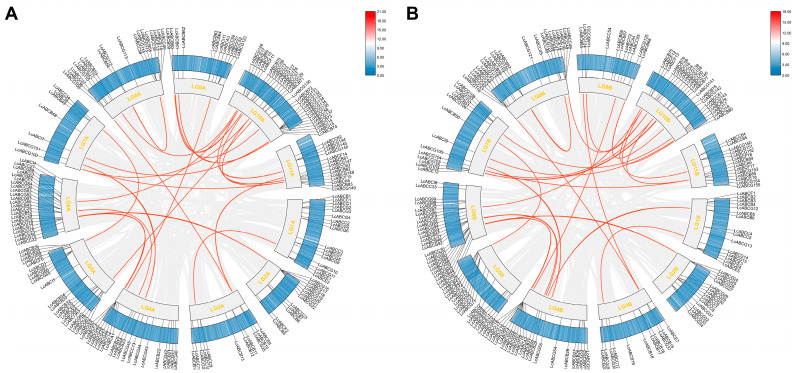
Homology analysis of *LcABC* genes in *L. chuanxiong*. (**A**) Intragenomic collinearity analysis of Haplotype A. (**B**) Intragenomic collinearity analysis of Haplotype B. The outermost black line marks the location of the *LcABC* gene on the chromosome. Red lines indicate homologous *LcABC* gene pairs in the genome. Concentric circles from inner to outer represent chromosome numbers and chromosomal density heatmap.

**Figure 3:**
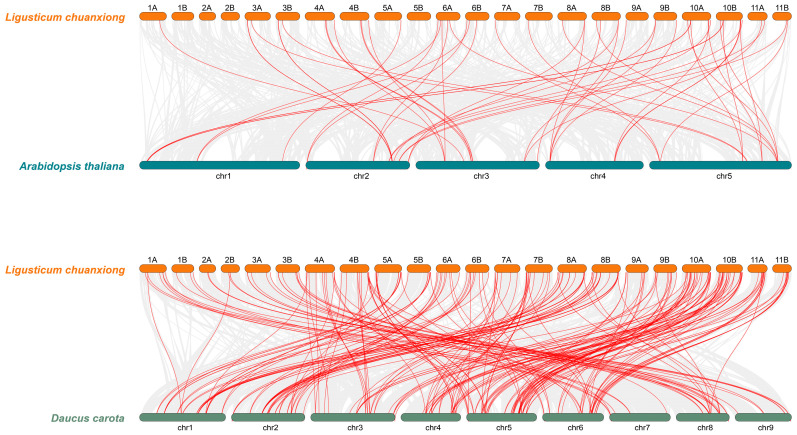
Comparative collinearity analysis between *L. chuanxiong* (turquoise blue) and *A. thaliana* (steel blue), and between *L. chuanxiong* and *D. carota* (lava orange). Red lines indicate collinear *ABC* gene pairs.

To investigate duplication events within the *LcABC* gene family, collinearity analysis was performed on all 368 genes across both haplotypes (Figure 2). Haplotype A contained 24 collinear gene pairs, while haplotype B contained 27 such pairs (Supplementary Table S2). Inter-genomic collinearity analysis was further conducted between *L. chuanxiong* and both *A. thaliana* and *D. carota*. This analysis revealed 48 collinear gene pairs between *L. chuanxiong* and *A. thaliana*, and 178 pairs between *L. chuanxiong* and *D. carota* (Figure 3; Supplementary Table S3). These results indicated a higher degree of genomic conservation between the two Apiaceae species, *L. chuanxiong* and *D. carota*; concurrently, 190 species-specific genes were identified in *L. chuanxiong*.

### 3.3. Differential Expression Analysis

To investigate the expression dynamics of *L. chuanxiong* under cadmium stress, RNA-seq libraries were constructed and sequenced from a total of 12 samples across four concentration groups, with three independent biological replicates assessed per concentration. Approximately 646 million 150 bp paired-end reads (65 Gb) were generated on the Illumina platform, corresponding to an average of 54 million reads per tissue sample. Read counts mapped to each gene were quantified using HTSeq (v0.9.1) as raw expression values. To ensure comparability of gene expression levels across different genes and samples, FPKM normalization was applied for standardization.

**Figure 4:**
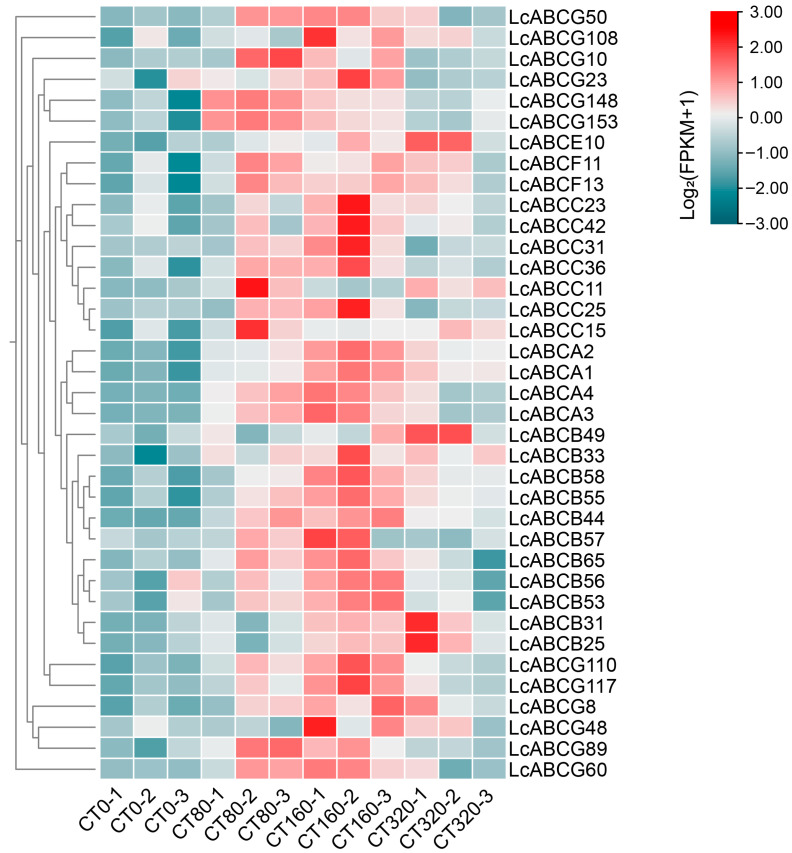
Expression patterns of 37 upregulated *LcABC* genes under cadmium stress. Heatmap was generated using log_2_(FPKM + 1) transformed expression values across various samples.

Based on the normalized gene expression matrix, we conducted a differential expression analysis to identify cadmium-responsive genes. First, differential expression analysis was performed for each cadmium treatment group (80, 160, and 320 mg/kg) separately against the single blank control (0 mg/kg) using the limma package V3.64.3 (thresholds: |log2FC| > 0.5 and *p*-value < 0.05). To provide a comprehensive view of the transcriptomic response to cadmium, we defined a union set of differentially expressed genes (DEGs) by combining all genes that exhibited significant expression changes in any of the three individual comparisons. This final non-redundant DEG set comprised 6522 up-regulated and 3549 down-regulated genes. Among these, 37 up-regulated differentially expressed *LcABC* genes were identified and are presented in a row-scaled heatmap (Figure 4).

**Figure 5:**
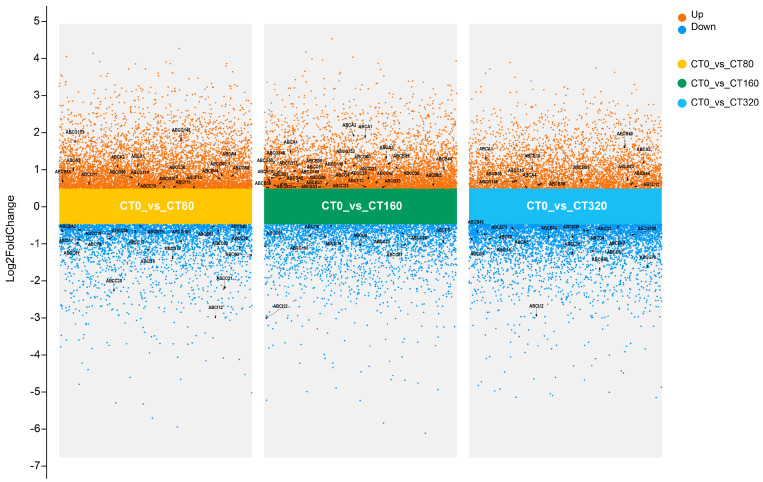
Multi-comparison differential volcano plot. Differentially expressed *LcABC* genes are highlighted with black arrows in the figure.

Additionally, to visually contextualize these specific genes within the global expression changes across all treatments, A multi-group volcano plot is included (Figure 5), highlighting the up- and down-regulated DEGs in each cadmium treatment versus the control, with the significantly expressed *LcABC* genes specifically annotated.

### 3.4. Phylogenetic Relationships

**Figure 6:**
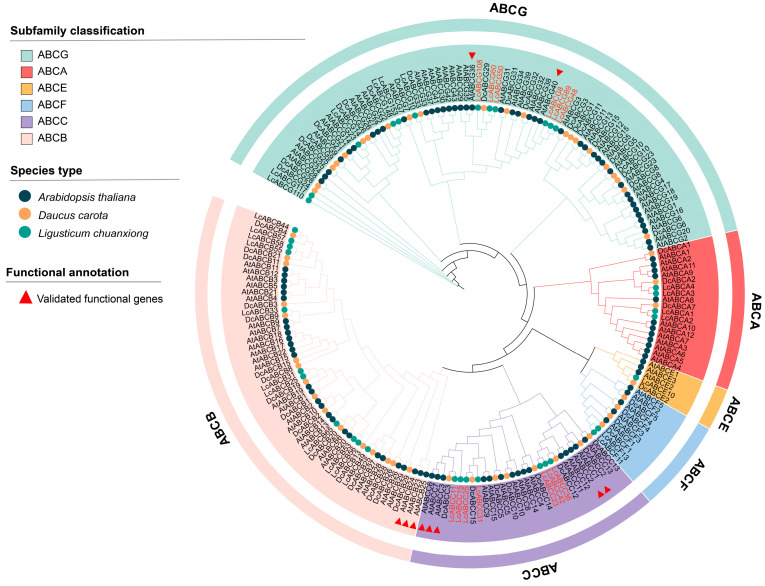
Phylogenetic tree of the 37 upregulated LcABCs with ABC transporter families from *A. thaliana* and *D. carota*. Different colors represent different subfamilies. The circle at the bottom of the branch indicates the species origin of the ABC protein: steel blue circles for AtABCs, lava orange circles for DcABCs, turquoise blue circles for LcABCs, and red triangles for functionally characterized AtABCs. LcABC transporter IDs clustering with the latter are highlighted in red.

To elucidate evolutionary relationships among the 37 targeted *LcABCs* and their orthologs from *A. thaliana* and the related species *D. carota*, we performed multiple sequence alignment using MAFFT and constructed a phylogenetic tree with the SMS model. The tree incorporated 10 previously reported AtABCs associated with heavy metal stress [33,34,35,36,37,38,39] (Figure 6). A total of 197 ABC transporters from *L. chuanxiong*, *A. thaliana*, and *D. carota* clustered into six subfamilies (ABCA-ABCC and ABCE-ABCG), showing high evolutionary conservation within each subfamily across species. A total of 13 LcABCs clustered closely with known heavy metal stress-responsive AtABCs, including AtABCC1/2/3/6/7, AtABCB23/24/25, and AtABCG36/40. Additionally, LcABCG117 and LcABCG110 exhibit long evolutionary branches.

### 3.5. Physicochemical Properties, Subcellular Localization, and Transmembrane Structure Prediction

Physicochemical analysis of the 37 up-regulated ABCs (Table 1) revealed that the number of amino acids encoded by these ABC transporters varied considerably, ranging from 171 to 1495. Molecular weights ranged between 19,447.17 and 169,147.6 Da, with theoretical isoelectric points from 5.53 to 9.57. The grand average of hydropathicity values spanned from −0.34 to 0.781, classifying 12 as hydrophilic proteins and 25 as hydrophobic proteins. Instability indices fell between 31.45 and 46.64, indicating 25 stable proteins and 12 unstable proteins. Aliphatic indices ranged from 78.29 to 108.89. Subcellular localization prediction (Supplementary Table S4) showed 29 genes localized to the plasmalemma, 3 to the cytoplasm, 2 to the nucleus, 2 to the endoplasmic reticulum, and 1 to the vacuole. Transmembrane structure analysis predicted that the vast majority of 37 LcABC proteins contained transmembrane domains; LcABCG117 and LcABCG110 were additionally predicted to possess signal peptides. Five LcABC proteins were predicted to contain globular domains.

### 3.6. Gene Structure, Conserved Domains, and Motif Analysis

**Figure 7:**
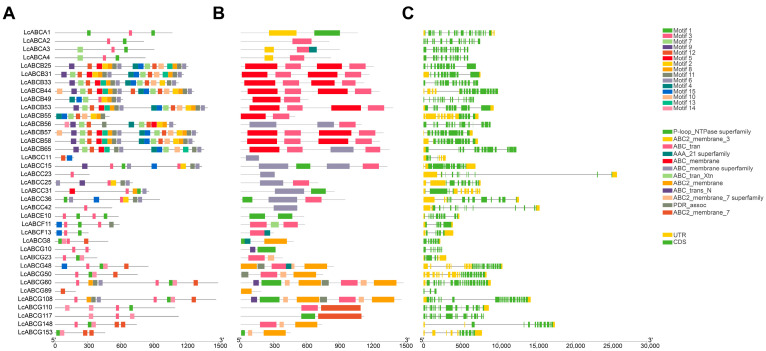
Schematic representation of conserved motifs, domains, and gene structures. (**A**) Prediction of 15 conserved motifs represented by colored boxes. (**B**) Conserved domain analysis. (**C**) Gene structures: coding sequences (CDS) shown in green rectangles, untranslated regions (UTRs) in yellow rectangles, and introns as black lines.

Systematic analysis of gene structures, conserved motifs, and conserved domains provided valuable insights into the similarities and diversities among these 37 up-regulated differentially expressed genes under cadmium stress. Conserved motif analysis of the 37 LcABC protein sequences using MEME suite revealed substantial variations in the number and arrangement of conserved motifs (Figure 7A, Supplementary Table S5). All members contained the relatively stable Motif3-Motif1 structure. Compared to other subfamilies, ABCB members exhibited more complex motif compositions, all containing both the (motif 7 + motif 12) and (motif 8 + motif 11 + motif 6) combinations. Conserved domain analysis using NCBI Batch CD-Search demonstrated highly similar domain architectures across subfamilies (Figure 7B). The ABC_tran domain was present in the vast majority of genes and frequently co-occurred with transmembrane domains. Gene structure analysis of the 37 *LcABC* genes was performed using TBtools V2.056 [25] based on the *L. chuanxiong* annotation GFF data, showing considerable variation in coding sequence length among these genes (Figure 7C). Members of the ABCA subfamily particularly exhibited significant lack of non-coding regions.

### 3.7. Prediction of Promoter Cis-Acting Elements

**Figure 8:**
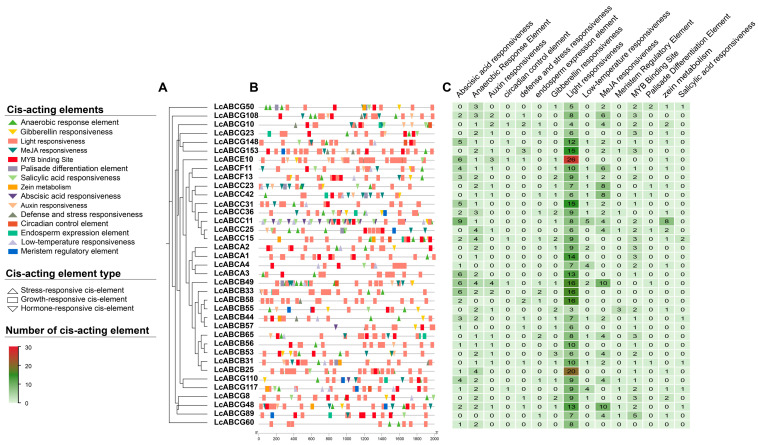
Cluster analysis and promoter cis-acting element prediction of 37 *LcABC* genes. (**A**) Clustering of 37 *LcABC* genes. (**B**) Visualization of cis-acting element distribution in 2000 bp promoter regions, where colored elements represent different functional types: upright triangles for stress-related elements, rectangles for growth/development-related elements, and inverted triangles for hormone-related elements. (**C**) Number of cis-acting element elements.

Sequences 2000 bp upstream of the prioritized 37 *LcABC* genes were extracted and analyzed for the prediction of cis-acting elements in the promoter region (Figure 8). The prediction revealed diverse cis-acting elements, broadly categorized into stress-responsive, hormone-responsive, and growth/development-responsive elements. These included light-responsive, auxin-responsive, gibberellin-responsive, low-temperature-responsive, salicylic-acid-responsive, anaerobic-induction, auxin-response, circadian-regulation, seed-specific-regulation, endosperm-expression, wound-response, and drought-induction elements. Among these, light-responsive elements were most abundant, totaling 372 occurrences, and were present in all 37 *LcABC* gene promoters. Notably, ten genes, including *LcABCG48*, *LcABCG108*, *LcABCB31*, *LcABCB44*, and *LcABCB58,* contained stress-responsive cis-acting elements in their promoter regions.

### 3.8. Analysis of LcABC Gene Expression Patterns

**Figure 9:**
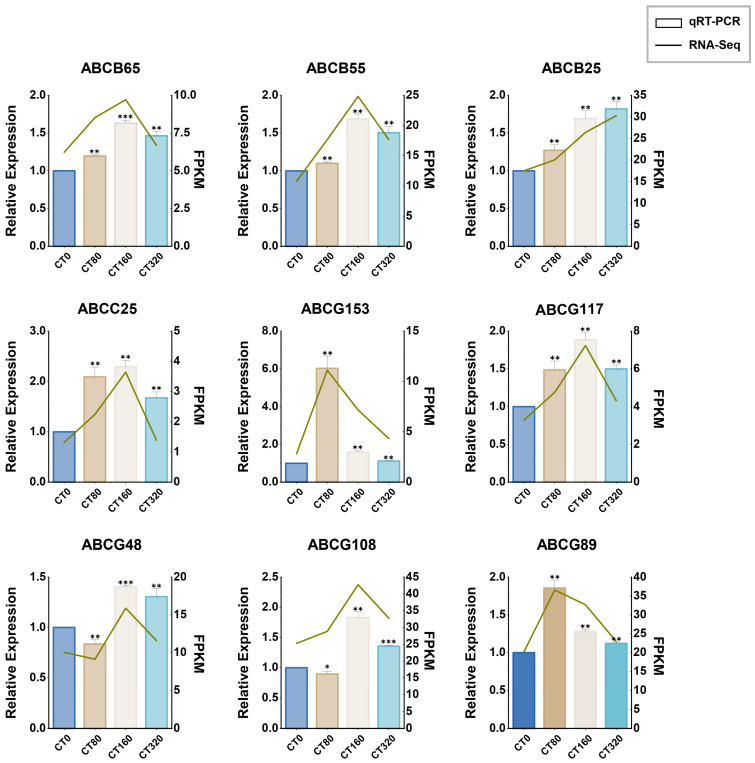
Expression profiles of nine *LcABC* genes under cadmium stress treatments. The *X*-axis represents different cadmium concentrations applied to *L. chuanxiong* plants, the left *Y*-axis indicates relative expression levels of the genes and the right *Y*-axis shows gene expression levels in FPKM from RNA-seq data. Asterisks indicate significant differences between treatments: * *p* < 0.05, ** *p* < 0.01, *** *p* < 0.001.

The expression patterns of nine *LcABC* genes (*LcABCB65*, *LcABCB55*, *LcABCB25*, *LcABCC25*, *LcABCG153*, *LcABCG117*, *LcABCG48*, *LcABCG89*, and *LcABCG108*) under different cadmium stress concentrations had been analyzed via qRT-PCR, with their expression profiles visually integrated with RNA-Seq data (Figure 9), though distinct genes showed specific responses to different cadmium concentrations: *LcABCB55*, *LcABCB65*, *LcABCC25*, *LcABCG48*, *LcABCG117*, and *LcABCG108* displayed the highest sensitivity to moderate cadmium concentration (160 mg/kg), *LcABCG89* and *LcABCG153* reached peak expression under low-concentration stress (80 mg/kg), whereas *LcABCB25* achieved maximal expression under high-concentration extreme stress (320 mg/kg).

## 4. Discussion

ABC transporters have been systematically identified and characterized in model plants and important crops such as *Arabidopsis thaliana* [40], *Zea mays* [41], *Solanum lycopersicum* [42], *Oryza sativa* [43] and *Triticum aestivum* [44]. However, the structural characteristics and functions of the *ABC* gene family in *L. chuanxiong* remain unclear. This study presents the first systematic identification of the *ABC* gene in the Apiaceae medicinal plant *L. chuanxiong*. By exposing *L. chuanxiong* to varying concentrations of cadmium stress and conducting transcriptome analysis, we identified key genes involved in the cadmium stress response. A comprehensive analysis of their physicochemical properties, structural features, and expression patterns was performed. These findings provide valuable insights into the genetic and molecular mechanisms underlying cadmium tolerance in *L. chuanxiong*.

A total of 368 *ABC* genes were identified in the *L. chuanxiong* genome and classified into eight subfamilies. Chromosomal localization revealed an uneven distribution of these genes with extensive tandem duplication events, suggesting considerable diversification during the evolution of the ABC gene family in *L. chuanxiong*. Comparative synteny analysis identified 24 and 178 ABC gene pairs between *L. chuanxiong* and *A. thaliana* and between *L. chuanxiong* and *D. carota*, respectively. These results indicate that *L. chuanxiong* and *A. thaliana* have undergone extensive independent evolution since divergence from a common ancestor, while *L. chuanxiong* and *D. carota* retain broad chromosomal conservation. Additionally, 190 *L. chuanxiong*-specific *ABC* genes were detected, further supporting the unique evolutionary history of the *ABC* gene family in this species.

Based on transcriptome analysis of cadmium-stressed *L. chuanxiong* plants, 37 up-regulated ABC genes were selected as key targets for further investigation. Phylogenetic analysis clustered the encoded proteins into six major subfamilies: ABCA–ABCC and ABCE–ABCG. Among them, 13 LcABC transporters—including LcABCC15, LcABCC25, LcABCC11, LcABCG48, LcABCG89, and LcABCG108—exhibited high sequence homology with *A. thaliana* heavy metal stress-related transporters such as AtABCC1, AtABCC2, AtABCC3, AtABCG36, and AtABCG40, suggesting functional conservation and highlighting their potential as candidates for future research. Notably, LcABCG117 and LcABCG110 displayed long evolutionary branches, implying species-specific adaptive evolution within the heavy metal-enriched environment of *L. chuanxiong*.

Substantial variation was observed among these 37 *ABC* genes in coding sequence length, theoretical isoelectric point, grand average of hydropathicity, instability index, and aliphatic index, suggesting diversity in their active sites, structural stability, and substrate-binding characteristics. Gene structure analysis revealed a reduced number or complete absence of introns in some genes, consistent with previous findings [45], suggesting that compact gene structures may facilitate rapid transcriptional responses to external or internal stimuli [46]. Conserved motif analysis further indicated evolutionary conservation in sequence alongside variations in motif quantity and arrangement. The observation of tandem TMD-NBD duplication motifs in multiple genes is consistent with the established molecular mechanism of ABC transporters, which rely on ATP hydrolysis for transmembrane transport [47]. Subcellular localization prediction indicated that approximately 86.5% (32/37) of up-regulated differentially expressed LcABC transporters localized to the membrane system, consistent with their potential role in heavy metal ion transmembrane transport or efflux [48]. Promoter element prediction classified cis-acting elements into three major categories: growth and development, hormone response, and stress response, indicating diverse roles of the ABC gene family in *L. chuanxiong* growth processes. Consistent with research by Sapna and colleagues [49], which demonstrated that cadmium stress modulates hormonal signaling cascades and activates oxidative stress responses in *Brassica juncea*, cadmium stress likely induces *LcABC* gene expression by activating specific transcriptional regulatory networks such as hormone signaling or oxidative stress pathways, thereby enhancing cellular defense against heavy metal toxicity. These findings provide important clues for understanding the structural diversity and functional differentiation of the ABC transporter family.

Numerous studies have demonstrated important roles of the *ABC* gene family in abiotic stress responses. Substantial evidence has established the crucial involvement of ABC transporters in plant responses to abiotic stress. Previous research demonstrated that *ZmABCB1* expression was significantly enhanced in cold-tolerant maize lines under low-temperature stress conditions [50]. Similarly, studies in rice have reported that ABCG transporters play vital roles in disease resistance mechanisms [51]. Our analysis of nine *LcABCs* revealed significant up-regulation under cadmium stress, reflecting the crucial role of the *LcABC* gene family in cadmium stress response. This differential expression pattern clearly demonstrates that *LcABC* gene family members possess unique expression characteristics and potential functional specialization when responding to varying cadmium stress intensities.

Despite the identification of 368 *ABC* genes in *L. chuanxiong*, the transcriptomic response induced by cadmium stress was remarkably limited; only 37 genes were significantly upregulated (Supplementary Table S6), while 28 were downregulated (Supplementary Table S7). This phenomenon reveals the precise transcriptional regulatory strategy employed by plants under adverse conditions. ABC transporters are widely involved in various fundamental growth and developmental processes such as gametogenesis, seed development, and organ formation [52], suggesting that most *LcABC* genes may perform indispensable constitutive functions, requiring stable expression under stress to maintain basic physiological activities. Concurrently, defense responses against cadmium stress—a costly abiotic challenge—often lead to growth inhibition and depletion of energy reserves, thereby triggering a strategic reallocation of resources from growth to defense [52].

*L. chuanxiong* appears to have evolved a highly economical and specific induction of a core detoxification and environmental adaptation “toolkit”, represented by the 37 upregulated *LcABC* genes. Regarding the 28 downregulated *LcABC* genes observed under cadmium stress, we reasonably speculate an analogous mechanism to that in tomato, where pathogen infection or other stresses activate jasmonate-responsive genes to initiate defense responses. However, excessive activation of jasmonate signaling can substantially consume cellular energy and suppress plant growth and development. Therefore, MYC2 and MTB1-3 form an elaborate negative feedback loop to terminate jasmonate signaling [53]. Similarly, the downregulation of *LcABC* genes may be interpreted as such an adaptive strategy for resource optimization and interference avoidance—by repressing transport activities that are non-essential or potentially conflicting with the core cadmium detoxification pathways, the plant can concentrate limited resources on central defense mechanisms, thereby maximizing its survival fitness.

This study has conducted a genome-wide identification of the ABC transporter family in *L. chuanxiong* combined with transcriptomic analysis, laying a foundation for future dissection of the molecular mechanisms underlying ABC transporter-mediated responses to cadmium stress.

## 5. Conclusions

The research identified 368 *ABC* genes in *L. chuanxiong* through genome-wide analysis. Their uneven chromosomal distribution and frequent tandem duplication events reflect considerable diversification during the evolutionary history of this species. Based on transcriptome data from cadmium-stressed samples, 37 up-regulated genes were selected for further study. These key candidates belong to six subfamilies: ABCA–ABCC and ABCE–ABCG. Phylogenetic analysis revealed that several *LcABCs* are closely related to *AtABCs* known to be involved in heavy metal stress responses in *A. thaliana*. Notably, some of these genes, *LcABCG8*, *LcABCG48* and *LcABCG108*, were also found to contain stress-responsive elements based on cis-acting element prediction, highlighting them as key candidate genes for further experimental validation. Furthermore, varying sensitivity to cadmium concentration among different *LcABC* genes suggests functional specialization in response to differing stress levels. This work lays a foundation for understanding the role of the *ABC* gene family in cadmium tolerance in *L. chuanxiong*.

## Figures and Tables

**Table 1:** Physicochemical properties of 37 upregulated LcABCs.

Gene Name	Number of Amino Acid	Molecular Weight	Theoretical Isoelectric Point (pI)	Instability Index	Aliphatic Index	Grand Average of Hydropathicity
LcABCA1	1075	119,431.5	6.67	41.84	94.82	0.052
LcABCA2	814	90,103.25	7.16	35.97	92.41	−0.042
LcABCA3	909	101,255.2	6.97	36.39	88.18	−0.034
LcABCA4	828	91,857.45	6.21	32.42	92.91	0.023
LcABCB25	1222	133,333.4	8.44	42.23	101.06	0.128
LcABCB31	1181	128,654.1	8.66	41.14	102.92	0.161
LcABCB33	1133	122,246.4	8.67	35.75	106.88	0.203
LcABCB44	1275	137,489	6.19	33.09	102.89	0.139
LcABCB49	622	67,955.69	9.36	35.52	104.1	0.106
LcABCB53	1400	154,171.4	5.53	41.95	106.41	0.153
LcABCB55	499	54,213.21	7	31.69	92.06	0.083
LcABCB56	1110	123,111.7	5.92	44.83	100.77	0.072
LcABCB57	1312	141,687.2	6.45	35.9	103.19	0.157
LcABCB58	1280	138,043.1	8.06	34.36	98.16	0.115
LcABCB65	1367	151,502	5.84	46.64	99.2	0.039
LcABCC11	171	19,447.17	9.57	40.21	108.89	0.781
LcABCC15	1347	151,452.5	8.52	37.77	103.36	0.08
LcABCC23	315	36,824.43	9.41	44.38	98.06	0.131
LcABCC25	712	79,208.07	5.61	35.35	105.84	0.067
LcABCC31	862	96,749.3	8.2	43.24	107.34	0.142
LcABCC36	961	106,903.7	6.27	35.69	102.97	−0.01
LcABCC42	522	60,474.39	9.05	36.44	102.49	0.258
LcABCE10	582	65,502.82	8.1	40.69	90.22	−0.128
LcABCF11	590	66,144.15	6.62	36.8	90.32	−0.293
LcABCF13	305	33,734.84	7.65	41.28	92.52	−0.248
LcABCG8	486	55,251.95	5.91	31.51	94.92	0.331
LcABCG10	323	35,789.64	8.93	39.49	91.42	−0.34
LcABCG23	385	42,130.02	8.31	38.93	88.6	−0.247
LcABCG48	855	96,546.96	5.71	33.1	95.12	0.232
LcABCG50	757	85,451.88	8.36	35.09	90.29	0.076
LcABCG60	1495	169,147.6	7.92	35.24	93.12	0.008
LcABCG89	187	21,582.04	9.21	43.44	78.29	0.211
LcABCG108	1477	168,134.5	8.63	39.35	88.99	−0.053
LcABCG110	1101	122,947.2	9.06	36.55	88.48	−0.217
LcABCG117	1133	126,118.7	8.7	37.53	87.94	−0.15
LcABCG148	748	82,638.31	8.3	34.7	95.31	−0.011
LcABCG153	458	51,492.03	7.69	31.45	105.37	0.237

## Data Availability

The datasets used and analyzed during the current study are available from the corresponding author on reasonable request. However, most of the data are shown in the Supplementary Materials.

## Supplementary Materials

The following supporting information can be downloaded at: https://www.mdpi.com/article/10.3390/genes16101235/s1, Table S1: qRT-PCR primers for *LcABCs*; Table S2: Gene duplicated *LcABC* genes; Table S3: Syntenic relationships of *LcABC* genes with other species; Table S4: Subcellular Localization and Transmembrane Domain Analysis of the 37 upregulated ABC Transporters; Table S5: Specific conserved motifs identified by MEME among 37 upregulated LcABC proteins; Table S6: upregulated LcABC protein sequences; Table S7: downregulated LcABC protein sequences.
